# Charting a Path to Success in Virtual Screening

**DOI:** 10.3390/molecules201018732

**Published:** 2015-10-15

**Authors:** Stefano Forli

**Affiliations:** Molecular Graphics Laboratory, Department of Integrative Structural and Computational Biology, The Scripps Research Institute, 10550 North Torrey Pines Road, La Jolla, CA 92037, USA; E-Mail: forli@scripps.edu; Tel.: +1-858-784-2055; Fax: +1-858-784-2860

**Keywords:** docking, drug design, molecular modeling, virtual screening

## Abstract

Docking is commonly applied to drug design efforts, especially high-throughput virtual screenings of small molecules, to identify new compounds that bind to a given target. Despite great advances and successful applications in recent years, a number of issues remain unsolved. Most of the challenges and problems faced when running docking experiments are independent of the specific software used, and can be ascribed to either improper input preparation or to the simplified approaches applied to achieve high-throughput speed. Being aware of approximations and limitations of such methods is essential to prevent errors, deal with misleading results, and increase the success rate of virtual screening campaigns. In this review, best practices and most common issues of docking and virtual screening will be discussed, covering the journey from the design of the virtual experiment to the hit identification.

## 1. Introduction

The goal of a virtual screen is to provide chemical insights and inspirations for designing a drug [1]. Automated docking of small molecules is applied in many drug design efforts, and due to the ubiquitous availability of large computational resources, high throughput virtual screenings are now routinely applied in campaigns to assess druggability of novel targets where often no binders are known. A large number of successful applications have been reported using a variety of docking techniques [2,3,4,5,6,7,8,9,10,11]. However, despite the improvements in recent years, a number of issues remain unsolved. The binding event is a fairly intricate process which imposes at the same time dramatic and subtle changes on the components forming the complex, where ostensible conformational changes are associated with invisible perturbations in the energetic equilibria. Modeling this event, where enthalpy and entropy components are deeply entangled, is a non-trivial task that can require a considerable computational effort. Indeed, most of the challenges faced when running docking experiments are due to the simplified approaches applied to achieve high-throughput speed and evaluate large numbers of ligands. Being aware of approximations and limitations of the method is essential to prevent errors, deal with misleading results, and increase the success rate of virtual screening campaigns.

**Figure 1:**
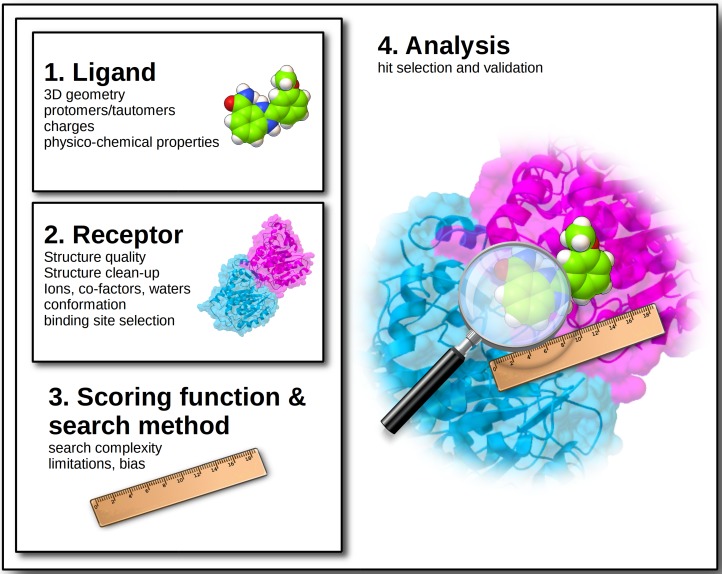
Overview of the main issues to be addressed when designing a virtual screening experiment.

At the dawn of computer history, Charles Babbage, the creator of the first programmable computer was asked: “*Pray, Mr. Babbage, if you put into the machine wrong figures, will the right answers come out?*” [12]. Almost two centuries later, the answer is still the same: starting from wrong data will inevitably compromise the results, regardless of the accuracy of the calculation performed. Therefore, the very first rule of any scientific simulation should be to ensure the highest possible quality of input data, which, specifically for docking, refers to ligand and target input structures. This will not guarantee success by all means, but will definitely reduce chances of failure. Best practices and most common issues of docking and virtual screening will be discussed, covering the journey from the design of the virtual experiment to the hit identification (Figure 1).

## 2. Ligand Structures

The choice of the library of ligands to be screened is one of the most stringent criteria, *de facto* determining the chances of finding a potential binder for the target of interest. The following guidelines should be considered when generating in-house virtual libraries, while the use of repositories such as ZINC [13] will provide high quality ligand structures ready to be docked.

### 2.1. Accurate 3D Geometries

In order to cut computation times, the vast majority of docking programs do not alter bond angles or bond lengths during the calculation, but sample only torsion angles. For these reason, it is a matter of paramount importance to generate proper input geometries with optimal bond angles and lengths (although, in some cases ligands may present distorted geometries when bound to enzymes, which specifically induce structural strains to facilitate the chemical reactions they catalyze) [14]. Beside energy minimization of the coordinates, it is also important to handle the absolute chirality of the stereogenic centers properly, or enumerate all enantiomers whenever it is not specified. This is often the case when a ligand library is generated from SMILES strings available from chemical vendors. Conformationally challenging ligands can also be modeled by pre-generating a large number of conformations to be docked rigidly [15]. Ring conformations, and macrocycles in particular, also need dedicated pre-processing because their conformation cannot be sampled effectively during docking. In this case, it is common to use protocols to generate multiple low-energy conformations prior to docking [16,17] and dock them independently; alternatively, specialized methods to simulate their flexibility during docking are available [18]. Several excellent tools have been developed over the years to generate high-quality coordinates, and many of them are available for free for academics. There can be used as standalone programs, such as OpenBabel [19] (and the Avogadro GUI [20]), RDKit [21], ChemAxon [22] or accessed as web servers, such as Frog3D [23], CACTVS [24]. For an excellent review on freely available conformer generators, see reference [25]. Finally, before accepting a ligand for a docking, it is important to check that the docking software to be used has parameters and proper atom types for each ligand atom. Some programs refuse to run if unsupported types are found, while other could silently assign a default type (with resulting low-quality parameters) and issue only a warning message in the log files. If the unparametrized element is essential to establish interactions with the target structure this can be a major issue. In fact, even in case of a successful docking run, the interaction energy could likely be not estimated correctly. There have been cases reported where wrong input geometries [26] generated very reactive species (accordingly to Sayle’s definition) [27].

### 2.2. Tautomers and Protonation States

**Figure 2:**
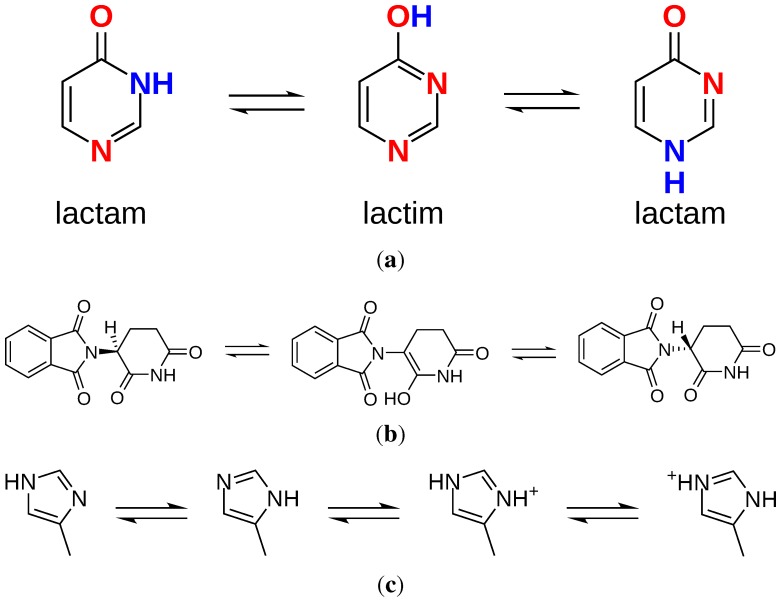
Examples of the effects of tautomerization and protonation states on molecular structures. (**a**) Tautomers of pyrimidin-4-one and hydrogen bond patterns [30] (*red*: acceptor; *blue*: donor); (**b**) Racemization of thalidomide via keto-enol tautomerisation [31]; (**c**) Histidine tautomers and protonation states [27].

The definition of tautomers as “isomers of organic compounds that readily interconvert, usually by the migration of hydrogen from one atom to another” [27] describes exactly what the vast majority of docking programs does not: account for changes in hybridization or hydrogen count on the ligand during docking. Specifically, the movement of hydrogens around a molecule (prototropic tautomerism) depends on several conditions such as pH, solvent and temperature [28,29]. One of the consequences of prototropic tautomerism is the dramatic modification of the hydrogen bond pattern (Figure 2a), or the change of chirality (Figure 2b). These events can determine the (in)success of a docking if (mis)matching with the target counterpart (Figure 2c).

The same can be true for protonation states. Depending on the pH of either experimental or physiological conditions being simulated, chemical moieties on the ligand can be protonated or deprotonated, acquiring charged groups that are not present in the neutral gas phase form of the molecule. In general, predicted stable protomers are generated at physiological pH 7.4, but catalytic sites might present micro-conditions which can deviate considerably from this value. This is often due to either the presence of acid residues (like those found in HIV-1 protease [32] or BACE-1 [33] sites) or basic metals like zinc (as found in carbonic anhydrase [34] or HDAC enzymes [35]).

**Figure 3:**
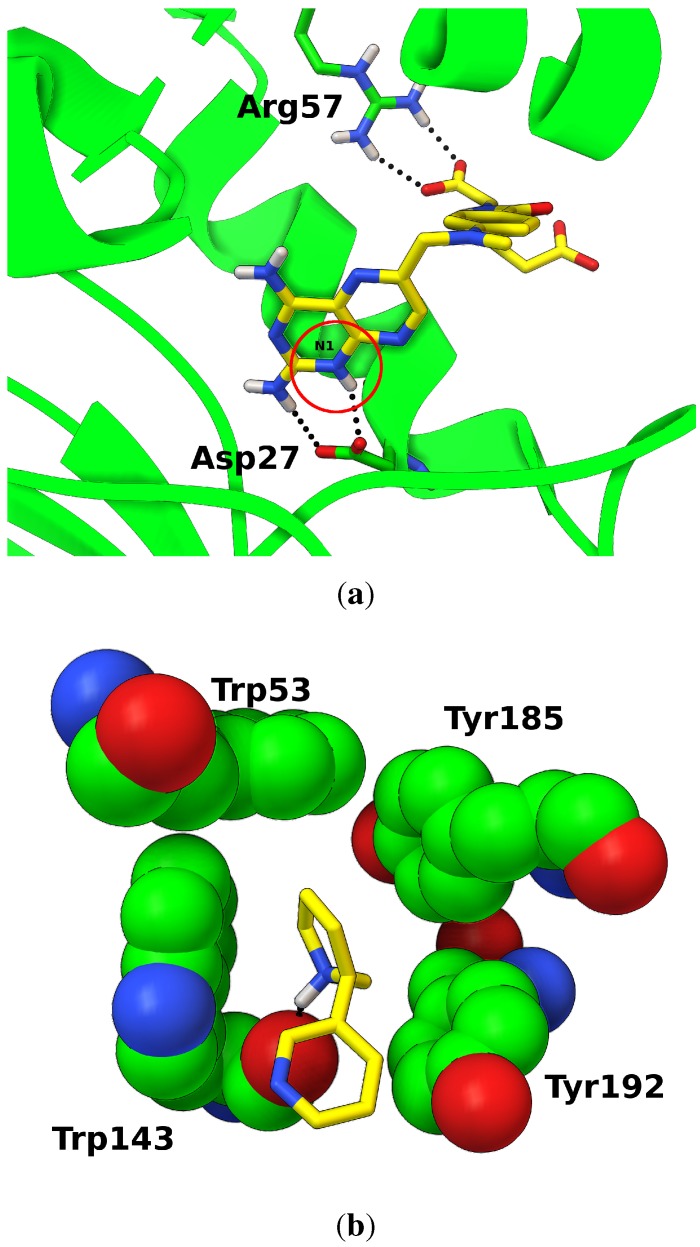
Examples of specific protonation states in ligand binding. (**a**) Methotrexate bound to *E.* coli DHFR; protein structure (*green*) is shown as secondary structure ribbons, and interacting residues as sticks; the ligand is shown as sticks (*yellow*); hydrogen bonds are shown as spheres (*black*); protonated N1 nitrogen is circled in red. (PDB:2inq) [36]; (**b**) Protonated nicotine bound to the acetylcholine binding protein (AChBP) of *L. stagnalis*. The ligand is shown as sticks (*yellow*); residues forming the aromatic cage are shown as spheres (*green*); hydrogen bonds are shown as spheres (*black*) (PDB:1uw6).

In some well-studied cases, experimental data or accurate calculations demonstrated the key role of tautomers and protonation states in ligand binding (Figure 3). In the case of the anticancer drug methotrexate, for example, neutron diffraction studies [36] identified which of the several stable protomers [37] is the most probable in the complex with DHFR: the two ligand carboxylic groups are deprotonated, while N1 nitrogen must be protonated to establish two hydrogen bonds with the aspartic acid (Figure 3a). If wrong protomers are docked (e.g., N1 is not protonated), the full hydrogen bond interaction network would not be restored, preventing the identification the correct docked pose or resulting in inaccurate score.

Another example is the positively charged nicotine bound to nicotinic acetylcholine receptors (nAChR) [38]. The charged feature of the the pyrrolidine ring nitrogen is an essential component of one of the oldest pharmacophores studied [39]. The receptor structure has a characteristic aromatic cage (Figure 3b) specialized in interacting and stabilizing the cation moieties in endogenous ligands.

Computational methods are available to predict tautomers and protomers [30], but their success is limited by the lack of availability of large experimental datasets on tautomer distributions. Moreover, enzyme binding is known to stabilize rare tautomers in ligands [30] that would be unlikely to be predicted. To date, ensemble dockings have been proven to be the most effective way to model multiple tautomers and protomers [40].

### 2.3. Charges

Charges are another aspect of ligand-receptor interaction that require special care. The formal charge of a molecule is a function of the chemical moieties it contains and the environment pH. In the context of a receptor structure, different local conditions that deviate from physiological conditions can induce protomers with different net charges than the ones predicted in solution.

Once formal charges are assigned, partial charges can be calculated. Partial charges are a simplified representation of the distribution of electrons in a molecule across its single atoms, and are the only way to model polarity (*i.e.*, dipoles) in classical force field simulations [41] (with the exception of computationally expensive and intricate polarizable force fields) [42,43].

Partial charges can be calculated using quantum mechanics (QM) [44,45,46], semi-emipirical [47,48,49], force field-based [50,51,52], or fully empirical [53] methods. QM and semi-empirical methods are the most accurate but also the most computationally demanding. Moreover, charges calculated with these methods depend on the molecular conformation, so several protocols have been proposed to overcome this limitation [54,55,56]. Force field-based or fully empirical methods, on the other hand, are orders of magnitude faster, conformationally independent, and provide acceptable results for most drug-like molecules. They are not suitable for inorganic compounds, metal-coordinating species and unstable/radical moieties, for which they lack appropriate parameters.

In principle, the choice of which method to use should be evaluated on the basis of the target considered, especially for systems where charge distribution is known to play a role [57,58]. However, scoring functions are usually calibrated using a specific charge set, therefore a consistency criterion should prevail (unless specific target properties cannot be properly described). Charges calculated with different methods will present a fairly large variability, and inevitably can have an influence on the results [59]. If different charge sets are applied to ligand and target structures, mismatching charge pairing can occur, likely increasing the false negative hit rate. Moreover, the absolute value of formal charges in large ligands should be assessed prior to docking because in strongly charged molecules, the Coulomb contribution to the scoring function will likely overcome other weak interactions, and increase the rate of false positives [55]. Most docking scoring functions cannot handle such highly charged systems unless specifically calibrated for that purpose. Conversely, it must be noted also that there are scoring functions which ignore entirely the electrostatic terms [60].

### 2.4. Physicochemical Properties

Intrinsic ligand properties can be used to pre-filter libraries to enrich them and limit the number of compounds to dock. One of the most popular criteria is the “rule of five” (Ro5) derived by Lipinski *et al.* [61] from a retrospective analysis of more than 2000 drugs. A similar filter, namely the ’rule of three’, was suggested for fragments with thresholds adapted to their smaller size [62]. Both correlate the number of hydrogen bonds, molecular weight, and octanol-water partition coefficient (logs*P*) to oral bioavailability and drug likeness. While criticism [63,64] from their indiscriminate enforcement as literal “rules” is well-deserved, their use as guidelines is recommended to provide support in prioritizing hits.

Another useful criterion to analyze a library is the identification of Pan Assay Interference Compounds (PAINS). A series of chemical groups has been found to be responsible for the promiscuity of certain compounds in many high throughput screenings [65]. Compounds containing these groups have been reported to be found in a suspiciously large number of assays, therefore they should be at least flagged as problematic, if not removed. More stringent criteria should be applied when filtering the library to exclude compounds containing reactive groups (e.g., acrylamide, chloroacetamide), which would establish undesired irreversible interactions with the target structure.

The size of a library, and with it the computation time, may also be reduced by using a diversity subset [66] on the assumption that similar ligands bind in a similar fashion. Hits obtained by screening the diversity set can be then used to generate a focused library containing ligands structurally similar to the hits, and this library can be used to sample the local chemical space for improved hits. A limitation of the approach is that the aforementioned assumption is not always valid. In tight cavities, for example, where shape complementarity criteria are more stringent, valuable results can be easily missed. Fragment-based drug design (FBDD) [67,68] is another efficient approach that provides high coverage of chemical space with relatively small libraries (1k–10k compounds) [68], often with relatively high hit rates [68]. Fragments are defined as small molecules with MW of ≤300 Da [69]. (compared to drug-like molecules with MW around 500 Da). In virtue of their small size, fragments are likely to require fewer structural adjustments on the target counterpart, therefore their binding can be easier to model. The main limitations of this approach are the issues of hit detection (see Section 5), the strong role of waters in fragment binding (see Section 3.4), and the added complexity of growing hit fragments into drug-like molecules [70].

Information about the target can also be used to tailor a more focused library. For example, if the library is built by strictly adhering to drug-like properties (*i.e.*, MW ≥ 450 Da.), it is unlikely to succeed in screenings on targets with very small pockets [71]. Conversely, very large binding sites are able to bind larger ligands, accommodating multiple binding modes [72] or even more than one ligand at the same time [73]. These structures are unlikely to be targeted effectively by libraries containing mostly fragment-like molecules.

## 3. Target Structure

The second crucial factor necessary for the proper outcome of a virtual screening campaign is the selection of the target structure to be used. Different criteria can be used to guide this selection, especially with structures obtained by crystallography [74,75].

### 3.1. Structure Quality

For structures obtained via X-ray crystallography, R-factor and resolution can provide an indication on the overall quality (low R-factor and high resolution should be preferred), but they do not provide details about specific regions [75]. More accurate information for single atoms or groups of atoms can be obtained by checking the B-factor (or temperature factor, or Debye-Waller factor) [76], which provides a measure of their dynamic behavior within the crystal (essentially, the lower the value, the lower the uncertainty on atom positions). Occupancy is another property used to define the quality of atom positions, providing a measure of the consistency of placement across the molecules in the crystal. Side chains that are solvent-exposed or that sample more than one conformation tend to have low occupancy values. In cases when the structure presents disordered regions (*i.e.*, regions where there is not enough electron density information to build the atomic models) the final structure could present either incomplete features (*i.e.*, sequence gaps, missing residues or side chains), or residues that are modeled in ’reasonable’ conformations, but have no experimental data to support them [75]. This modeled data is usually reported in the paper describing the structure and should be easily recognizable by its very high B-factor, but this is not always the case. Conversely, some regions of the density maps could be left uninterpreted, with undefined density blobs that can correspond to waters, crystallization buffer molecules, or even unidentified ligands [75]. Cases have been reported in which an unclear combination of human errors and software issues led to highly inaccurate structures [77,78]. When no experimentally determined structures are available, it is possible to use homology modeling techniques [79] to build reasonable models of the desired protein, provided that enough template structures with high sequence and structure homology are available. The degree of success in using these structures for structure-based drug design is variable and depends strongly on the specific type of proteins considered [80,81,82].

### 3.2. Structure Clean-Up

No structure should be used directly in a docking calculation without a certain degree of prior inspection and clean-up. Structures should be analyzed to check for the presence of artifacts such as improper bond lengths and angles, and overall conformation assessment of the backbone and side chains should be performed.

**Figure 4:**
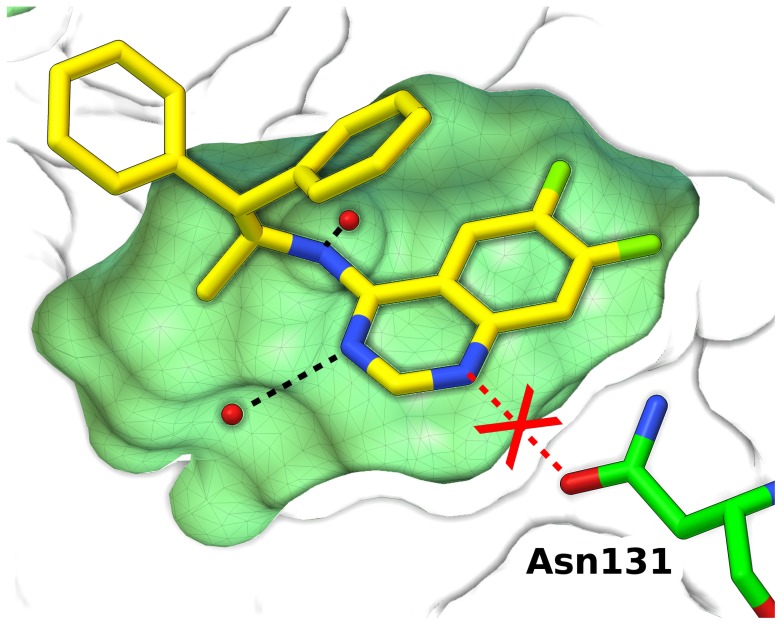
Wrong conformation of Asn131 side chain in the crystal structure of a scytalone dehydratase inhibitor prevents the formation a key hydrogen bond. Protein buried cavity is shown as surface (*green*); ligand (*yellow*) and Asn131 (*green*) are shown as sticks; waters are shown as spheres (*red*). Hydrogen bonds are shown as dashed lines (*black*) (PDB:5std) [84].

The amount of pre-processing required depends on the quality of the structure and the experimental conditions used for resolving it. Structures resolved by crystallography often contain salts and other molecules from the buffer, or additives used to induce the crystal formation. All these molecules should be removed prior to docking. Also, even in structures with acceptable quality, a certain degree of uncertainty is associated with the orientation of asparagine, glutamine and histidine side chains, since electron density is not sufficient to resolve the difference between nitrogen and oxygen atoms [83]. In the absence of structural hints suggesting the preference of one conformation over the other, their crystallographic conformation should not be trusted completely. Flipped conformations can also be assigned if the ligand structure is not considered during the structural refinement (see Figure 4). Structures solved via NMR usually contain an ensemble of models, so a single model should be extracted and processed separately. Structures obtained by neutron diffraction [85] usually have resolution high enough to distinguish hydrogen atoms. However, fully deuterated crystals (*i.e.*, all ^1^H atoms are replaced by ^2^H) [86] are often used to increase the signal-to-noise ratio and enhance the visibility of the molecular structure [87]. Many docking programs do not recognize deuterium properly, which has to be replaced by hydrogen when preparing these structures. The protein sequence used to solve the structures could differ from the wild-type or the one used during the assays. The reasons for such differences are various. Mutations can be inserted to study the insurgence of drug resistance [88,89] or inhibit the autolytic activity in an enzyme [90]. Non-natural amino acids can be inserted to address the phase problem in crystallographic structure determination [91]; recombinant proteins can be expressed using a medium containing selenium to replace the sulfur-containing residues cysteine and methionine with their corresponding selenium derivatives, selenomethionine [92] and selenocysteine [93]. In some cases, modifications can be substantial to the point where the protein sequence is altered to include entire domains that are not present in the wild-type. An example is provided by the chimeric GPCR structures engineered to create soluble (hence crystallizable) versions of the transmembrane proteins [94]. These modifications should be reverted when required (*i.e.*, to study the active form of an enzyme), or accounted for otherwise. Whenever missing atoms or missing residues are encountered, structures should be amended and discontinuities filled or capped. Specialized tools [95,96] may be used to rebuild incomplete residues or even entire missing loops [97]. Over the years, efforts have been made to prevent issues with structural models deposited in the PDB [98] by designing dedicated validation software [99,100,101]. While these tools are now applied routinely to check new PDB entries, it would be a good practice to run them on input structures prior to docking to check for errors that could be introduced during the preparation steps.

### 3.3. Protonation States

Similar to what discussed for the ligand, protonation states and tautomers play an important role also in the target structure. For histidine side chains, for example, four states are possible (Figure 2c). When more than one key residue can present multiple states, modeling only the most probable ones for each residue is not sufficient. For example, simulations [102] and experimental evidence [103] suggest that when a ligand is bound to the two catalytic Asp25 in the asymmetric homodimer of HIV-1 protease [32], the two carboxylic groups present an asymmetric ionization. Analogous conditions are found in β-secretase (BACE1) [104], where the protonation state of the catalytic dyad formed by Asp32 and Asp228 is essential to screen for inhibitors [105] Dockings of known inhibitors against eight distinct states of the dyad show that different binders prefer different states [33].

To date, the best way to model state multiplicity in the target is to dock against protomer and tautomer ensembles.

### 3.4. Coordinating Metal Ions, Co-Factors and Waters

Special care should be taken when dealing with structural features such as catalytic metals, co-factors and structurally conserved waters. Enzymes in which metal ions play a catalytic role (metalloenzymes) are widely distributed in all cells and involved in a large number of metabolic processes [106]. The modeling of ligand-metal coordination is problematic for most scoring functions [107,108,109], due to the difficulty in describing the partial covalent bond nature of the interaction [110]. For this reason, when docking in systems containing catalytic metals, the best choice would be to use docking programs that have been validated on the specific metal ion to be considered [110,111], or have dedicated terms in the scoring function to describe metal coordination [112,113].

As a general guideline, if a co-factor is bound to the structure, (e.g., NADP, heme, …) it should be conserved in the target structure used for docking. If removed, it would leave a favorable cavity unoccupied: docking results will be biased toward it, but it is unlikely that any ligand would be able to actually engage it when competing with the affinity and the concentration of an endogenous binder. Exceptions are of course possible, as when ligands are designed to explicitly to compete with a natural substrate. An example is the drug chloroquine, which binds in the active site of lactate dehydrogenate of *Plasmodium* falciparum displacing the NADH cofactor [114]. Methods to model competitive binding by docking multiple ligands at the same time have been developed, but they have not been extensively tested [115].

For waters, the common practice is to remove them entirely from the structure and dock into a “dry” model [116]. However, this may not be the optimal choice when conserved waters are known to have direct involvement in ligand binding [39,117], which is particularly frequent with fragments [118]. Several docking programs handle waters explicitly during docking with different degrees of approximation [47,116,118,119,120,121,122]. Whenever information on the possible involvement of waters is available, these methods should be preferred, especially when structurally conserved waters (*i.e.*, very low B-factors) have been identified in the cavities considered in the docking. In the case of low resolution structures, where no waters have been resolved, predictive methods can be tested [118,123].

### 3.5. Structure Conformation

**Figure 5:**
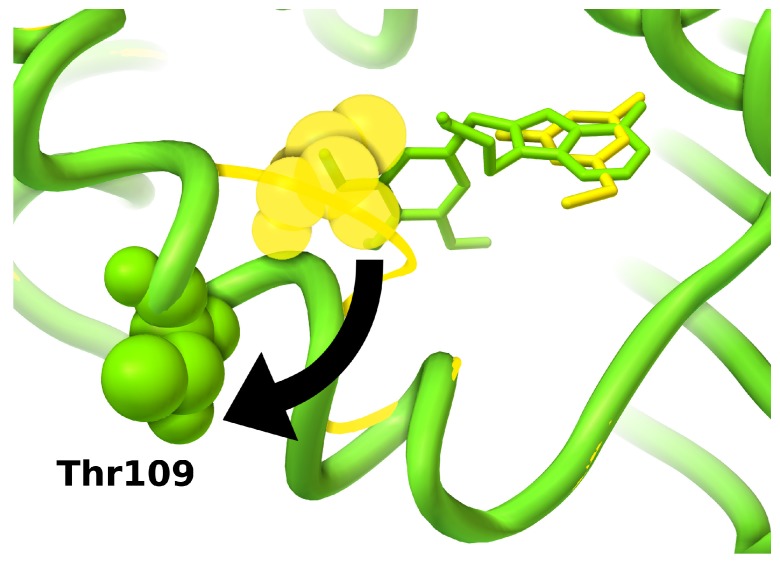
Conformational re-arrangements in HSP90 induced by ATP site inhibitors. Protein secondary structures are shown as ribbons; conformations of key residue Thr109 are shown as spheres; ligands are shown as sticks. Small fragments can bind into a protein conformation similar to the *apo* conformation (*yellow*, PDB:2wi2) [134], in which an unwinded loop covers part of the binding site; larger ligands induce conformational rearrangements folding the loop in a helix conformation and uncovering a larger binding site (*green*, PDB:1uy6) [131].

Proteins in solution exist in a variety of conformational states distributed in an complex energy landscape [124]. Some of these conformations represent discrete states which are associated with specific biological functions that have been extensively investigated, like the active/inactive states in protein kinases [125,126], and GPCRs [127,128,129]. On the other hand, the energetic components regulating the dynamic transitions between states is complex and still poorly understood [124,130]. Binding ligands can either stabilize existing conformations [125,128] or trigger specific conformational changes (Figure 5) [131]. Moreover, different ligands induce small re-arrangements in the binding site (induced fit) [132]. The scenario can be roughly summarized by saying that the receptor that binds with a predicted ligand is rarely what we expect it to be, so the choice of the structure to use must take in to account the structural variability of the target. In general dockings using *holo* structures provide higher enrichment rates than those performed using *apo* structures [133], likely because *holo* structures present some degree of induced fit due to the presence of a ligand.

It is important to take into account limitations and biases of the experimental method used to determine the structure. In X-ray crystallography, for example, structures are commonly solved at very low temperatures (100 K or lower) [135] and in conditions of supersaturation [136]. The formation of the crystal lattice during packing can add an extra bias, perturb backbone and side-chain conformations, and induce improper hinge-like motions [137]. Crystallographic conformations in general provide a biased representation of the flexible behavior of a protein. For example, for targets that display a lot of structural flexibility in solution, such as HIV-1 protease, a single NMR ensemble can present more structural variability than a hundred crystal structures [74]. Depending on the goal, a possible strategy could be to target highly structurally conserved regions [138], or focus on a well-defined specific conformation [125]. When this is not possible, conformational variability must be taken into account in docking experiments. There are different ways in which target flexibility can be modeled, as discussed in detail in recent reviews [139,140]. Essentially, two main classes of motions are modeled using orthogonal and complementary approaches: ligand induced-fit involving small and local re-arrangements of a few amino acids can be modeled with sufficient accuracy by simulating *flexible side chains* during docking; large conformational changes such as hinge and domain movements, loop rearrangements, and changes involving the protein backbone in general, can be modeled by running *ensemble dockings* on discrete pre-calculated conformations.

Depending on the software, the number of side chains that can be made flexible is limited by the increase in search complexity and exacerbation of scoring function limitations, such as simplified solvation models and unbalanced energy contributions between ligand and flexible receptor. Therefore, only a small set of residues should be made flexible, and the choice can be based on occupancy, B-factor values, or variability across multiple crystal structures (in complex with different ligands, if possible).

For ensemble dockings, multiple receptor conformations can be selected from experimental structures [141] or generated through computational methods such as molecular dynamics simulations [142], or normal mode analysis [143]. Ensemble docking has been reported to improve the overall success rate in virtual screenings [9,141,144,145,146], although requirements of computation time and power can increase considerably, scaling linearly with the number of target structures considered. Massive virtual screening campaigns, involving large conformational ensembles of flexible proteins, have been made possible with the support from IBM and the computer time donated by volunteers participating to the World Community Grid initiative [147].

### 3.6. Binding Site Selection

If a known binding site is going to be targeted, all available information should be used to define search boundaries. The search should encompass the regions occupied by known ligands, but not be limited to them, since alternative binding modes could engage residues that have not been characterized yet. However, too large search boundaries may reduce the accuracy, require longer computation times, and increase false positive rates. Known binders such as endogenous molecules should be added to the ligand library to be screened, especially if resolved in complex with the protein. Including them provides a validation of the screening protocol (“*are search parameters thorough enough to dock ligands similar to the known binder?*”) and the scoring function (“*can the scoring function recognize known binders as hits?*”). These docking results will also provide a score reference value to be used when evaluating the virtual screening hits.

When no binders are known, it is possible to run dockings considering the entire protein volume in a blind docking [148] experiment. While this method has shown a considerable degree of success [149,150], higher accuracy and efficiency can be achieved by running multiple focused dockings on regions predicted to have high affinity interactions [151]. In the identification of a site, any structural information available should be exploited. Crystallization additives and buffer molecules can provide insights [75] on potential interaction loci and preferred chemical moieties. Even structurally consistent waters (*i.e.*, with low B-factor and high occupancy) can hint at regions with favorable hydrogen bond interactions. A variety of software is also available to identify energetically favorable sites that are likely to accommodate ligands [152].

## 4. Scoring Function and Search Method

The scoring function is the key for a successful screening. A good scoring function should have an acceptable balance between speed and accuracy. Since the goal of a virtual screening is to identify new potential binders while reducing the number of unsuccessful assays, the ideal scoring function should also have a low rate of false positive hits. Scoring functions differ by their design and can be classified in three main categories: [153] empirical, knowledge-based and forcefield-based. Their quality is bound to the quality of the data sets used to build and calibrate them [75,154]. A great variability in success rate has been reported in comparisons between a number of docking programs [4,153,154,155] and the common agreement is that no software is the absolute best across all the targets. Extreme cases like strongly charged sites [156] or these with hydrophobic/hydrophilic dominant components [157] are known to be more problematic for general purpose scoring functions. Indeed, it has been suggested that generalized scoring functions should be replaced by target-specific models, which would likely yield better results [158]. Therefore, whenever possible, the scoring function should be validated for the particular target to be considered in the screening, using known ligands when available. Two main issues are common to most scoring functions. One is the additive nature of the score when estimating enthalpy. The different interaction terms composing a scoring function are calculated independently and the final score sums up these components. For this reason, larger molecules are likely to have higher scores because of their ability to establish more interactions [159] (see Section 5). The other is the poor description of the entropic component associated with losses in vibrational and conformational degrees of freedom [160].

The scoring function provides the description of the energetic landscape that the search algorithm will sample. The complexity of this landscape depends on the energetic terms composing the scoring function, and the degrees of freedom associated with any moving atoms in ligand and target structures. If the complexity gets beyond the capabilities of the search method, *i.e.*, very large and flexible ligands, too many flexible side chains, docking volume too large, *etc.*, the screening is bound to fail, or it will require a much larger computational effort to generate reliable poses. In order to maximize the chances of success, choices resulting in increased search complexity should be used with parsimony, while superfluous complexity should be avoided independently of the efficiency of the search algorithm. Conversely, any experimental data that could be used to simplify the search should be used. Finally, most of the search algorithms are stochastic [161], therefore, prior to running a full virtual screen, it is a good practice to test the search protocol with a representative subset of ligands from the library (even better: including ligands for which experimental coordinates are available). This will ensure that the protocol is robust enough, and that the search parameters are sufficient to guarantee a certain degree of convergence and consistency in the results.

## 5. Results and Assay

When docking calculations are completed, virtual screening results need to be processed and analyzed to identify a set of hits to be assayed. A series of criteria can be used to filter and prioritize results to be evaluated by either visual inspection or rescoring schemes.

### 5.1. Hit Selection

The goal of a virtual screening is to provide new chemical scaffolds as bioactive molecules for a given target [162], and a hit is defined as “*a primary active compound, with non-promiscuous binding behaviour, exceeding a certain threshold value in a given assay*” [163]. Screening hits are meant to provide as many and diverse starting points as possible for the identification of improved molecules (*i.e.*, leads), and the event of identifying of a high-affinity binder is rather unlikely, to the point that this expectation has been deemed as one of the pitfalls of virtual screening [162]. In fact, the accuracy of current scoring functions is known to be higher in reproducing the correct pose of a given ligand than in scoring multiple ligands [164]. Hence, some degree of chemical expertise can definitely help in the hit identification to discriminate between interesting compounds, reliable interactions, poses and predicted affinities. As a general guideline, achiral compounds are preferable to compounds with stereogenic centers: chiral compounds are in fact more complex to synthesize, while chemical vendors often provide only racemic mixtures (or charge extra for enantiomerically pure compounds). The first criterion for selecting hits is the rank (or score) provided by the docking, but it is rarely the only one. Ligand efficiency [165] calculated on the docking score can also be a useful metric to identify promising compounds [166], especially fragment-like molecules [167]. Ligand efficiency can also help mitigate the bias of scoring functions toward large ligands (see Section 4), because effective binders usually engage most of their atoms in specific high-affinity interactions. A common approach to prune results [168] is test different combinations of variable parameters (e.g., energy, ligand efficiency) and interaction filters to reduce the number of potential hits to a size that is suitable for visual inspection.

Knowledge-driven criteria should be considered whenever experimental data is available. Patterns such as interactions with key residues and chemical feature overlap with known binders [169] are useful to bias hit selection, while interactions with exposed backbone can be favored to avoid resistance from mutations [170].

Structural diversity should also be emphasized in order to sample a large portion of the chemical space. During the selection phase, experience and intuition (often in the form of rather arbitrary choices) [162] tend to compensate for a lack of absolute criteria for identifying hits, scoring function weaknesses, and model approximation.

Whereas relying on a single scoring function can bias the results, combining rankings generated with multiple docking programs (consensus scoring) has been used successfully to enrich results [171,172]. Also, once the number of potential hits has been reduced by orders of magnitude, it is possible to apply more computationally expensive scoring methods that provide better estimates of the free energy of binding by combining molecular mechanics and quantum mechanics [173], taking into account entropy terms and solvation effects [174,175,176,177,178] or using empirical methods [168].

### 5.2. Hit Validation

The caveat of any virtual screening shuold be: “ *Your computation is only as good as your experimental follow up*” (E.R. Zartler) [179]. Clearly, it is important that hits selected in the virtual screening are actually accessible for testing, either through commercial vendors or via feasible synthetic pathways. Ligand physico-chemical properties can affect the assay outcome. Among them, unpredictable poor water solubility is a common issue, while fluorescence can interfere with several common assays [180,181]. The choice of the assay itself can also impact the outcome of a screening, especially as a consequence of its minimum sensitivity threshold for low affinity binders. Therefore the choice of the assay should not overlook the size and expected affinity of compounds to be tested. This is particularly relevant for protein-protein interface inhibitors and fragments, which are expected to show relatively low affinities [182]. Interestingly, different experimental methodologies can identify different hits even when testing the same fragment library [183]. In general, assays that have been successfully applied and validated on the specific target should be preferred.

## 6. Conclusions

Virtual screening involves a large number of steps, and each step comes with its own pitfalls. While many issues are intrinsic to the simplified models used in the modeling, others are the result of improper pre-processing of the input or misinterpretation of the results. No single best recipe is available, and success rate may vary considerably depending on the target and the expertise of the user. Software is not (yet) meant to replace chemical intuition or deep knowledge of the biological target, which are essential to the identification of hits. Expertise is achieved over time, and as Niels Bohr said: “*An expert is a man who has made all the mistakes which can be made in a very narrow field*”. Luckily, thanks to software improvements and the massive computational power available today, it is possible to make in a few days the number of mistakes that not too long ago would have taken years.

Virtual screening is one of the many drug design tools available to scientists, and when properly used can be an invaluable support to research. Knowing how a method can fail is as useful as knowing how it works, so it is fundamental to maintain a critical attitude when dealing with results. A wise combination of rigorous approaches and clever workarounds can reduce chances of failure. Carefully pondering docking results and interpreting them as informed suggestions can significantly boost chances of success, and the rest is computation overhead.
